# Primary Actinomycosis of the Stomach: A Review of the Literature for A Rare Entity

**DOI:** 10.3390/jpm15030116

**Published:** 2025-03-17

**Authors:** Afroditi Ziogou, Ilias Giannakodimos, Alexios Giannakodimos, Evangelia Mitakidi, Nikolaos Charalampakis, Petros Ioannou

**Affiliations:** 1 Department of Medical Oncology, Metaxa Cancer Hospital of Piraeus, 185 37 Piraeus, Greece; aziogou@yahoo.com (A.Z.); alexisgianna@med.uoa.gr (A.G.); 2 Department of Urology, Laiko General Hospital of Athens, 115 27 Athens, Greece; iliasgiannak@med.uoa.gr; 3 Department of Anesthesiology, General Hospital of Nikea, 145 61 Piraeus, Greece; emitakidi@uth.gr; 4 Department of Internal Medicine, University Hospital of Heraklion, 71003 Heraklion, Greece

**Keywords:** actinomycosis, actinomyces, stomach, gastrointestinal tract, infection

## Abstract

**Background/Objectives:** Primary gastric actinomycosis is extremely rare and only a limited number of cases are published in the literature. Actinomycosis is caused by anaerobic Gram-positive bacteria; these microorganisms are members of the normal human microbiome and occasionally lead to infection, especially in immunocompromised patients or patients subjected to abdominal surgery. Advances in personalized medicine, including tailored antimicrobial therapy based on individual patient profiles, may enhance treatment efficacy and reduce unnecessary interventions. **Methods:** A review was performed through a literature search of the PubMed/MedLine and Scopus databases. **Results:** A total of 27 patients were included, 15 males (55.56%) and 12 (44.44%) females, with a mean age of 55.11 ± 17.48 years. Among the included patients, 25.93% had a history of abdominal surgery. Abdominal pain (73.08%), weight loss (40.74%), nausea or vomiting (30.77%) and fever (19.23%) constitute the most commonly reported clinical manifestations. Endoscopy (59.26%), computed tomography (48.15%), ultrasonography (22.22%) and magnetic resonance imaging (11.11%) assisted in indicating the primary lesion. Diagnosis was achieved preoperatively in 66.66% of patients, via endoscopy and biopsy (51.85%) or via cultures (14.81%), while nine cases (33.33%) were diagnosed postoperatively. The therapeutic approaches included antimicrobial administration (32%), surgery (24%) or both (44%). The most widely used antimicrobial was penicillin (77.78%) and the mean duration of antimicrobial treatment was 5.85 months. The protocol for this review was registered in Prospero (ID:CRD42025649532). **Conclusions:** Due to the divergent clinical presentation of primary gastric actinomycosis, clinicians should be aware of this rare entity in order to establish diagnosis in a timely manner and provide prompt and effective treatment.

## 1. Introduction

Actinomycosis constitutes a rare infection caused by anaerobic Gram-positive bacteria belonging to the genus of *Actinomyces* [1]. These bacteria normally colonize the human mouth, and digestive and genital tracts and, due to their low virulence, rarely result in active infection in immunocompetent individuals [2]. The incidence of actinomycosis is increased in immunosuppressed hosts, diabetic patients, and in patients with disrupted mucosa due to surgical procedures or trauma [3]. The cervicofacial region, the abdominopelvic region and the respiratory tract comprise the most commonly reported anatomic locations for the development of actinomycosis [1]. Gastric actinomycosis is an extremely rare entity and only a few case reports have been published in the literature [2]. These benign lesions mainly present with atypical symptoms and non-characteristic imaging features or endoscopic findings [4]. Their diagnosis remains challenging and mainly depends on the clinician’s high clinical suspicion; the ultimate diagnosis relies on histological findings, received either pre-operatively via endoscopic procedures or postoperatively, after surgical excision of the lesion [5]. Due to its atypical clinical manifestations, gastric actinomycosis is usually misdiagnosed, mimicking various conditions, such as malignancy, tuberculosis and nocardiosis [1]. Personalized medicine, incorporating molecular diagnostics and individualized antimicrobial regimens, may improve treatment outcomes by tailoring therapy to the patient’s specific microbiological and clinical profile. Possible therapeutic options consist of either preservative management, with prolonged administration of antimicrobials, or surgical intervention, aiming for surgical excision of the lesion [2]. The aim of the present study was to review the literature regarding primary gastric actinomycosis and highlight useful diagnostic modalities and optimal treatment of this rare entity.

## 2. Materials and Methods

This review aims to compile and analyze all documented cases of *Actinomyces* species infections affecting the stomach. Two independent researchers (A.Z., I.G.) conducted a systematic search of the PubMed/Medline and Scopus databases for relevant articles on gastric actinomycosis published up until January 30, 2025. The review protocol was registered in PROSPERO (ID: CRD42025649532). The search strategy utilized the following keywords: (“Actinomycosis” OR “*Actinomyces*”) AND (“gastric” OR “stomach”). In cases of disagreement between the investigators, a senior researcher (P.I.) was consulted for resolution. This study included case reports and case series describing *Actinomyces* spp. infections originating in the stomach, with no restriction on patient sex. Only studies published in English were considered. To ensure a focus on detailed individual cases, eligible articles were limited to case reports and case series. Review articles, systematic reviews, and retrospective studies without case-specific data were excluded. Additionally, articles lacking original or sufficient data, studies involving animals, comments, and publications categorized as “epub ahead of print” were not considered. Any articles that were inaccessible were also excluded. A supplementary manual search was performed using the reference lists of selected studies, following a snowballing approach, to identify any additional relevant publications. Three independent reviewers (A.Z., I.G., A.G.) extracted data autonomously. A standardized template was used to collect information on patient demographics, epidemiology, clinical presentation, and medical history. Furthermore, details related to diagnostic methods, treatment strategies, and clinical outcomes of gastric actinomycosis in humans were systematically gathered and analyzed.

### Statistical Analysis

Descriptive statistics were applied to numerical variables, with mean values and their corresponding standard deviations (SD) reported, or medians with interquartile ranges (25–75%) when data distributions were skewed. Categorical variables were summarized using frequencies and percentages to provide a comprehensive overview. Due to discrepancies in reporting across studies, not all outcomes of interest were consistently documented. Rates were calculated based on available data to offer a thorough synthesis of the findings. No statistical relationship between the included variables was tested. Statistical analysis was performed using IBM SPSS Statistics for Windows, Version 24.0 (Armonk, NY, USA: IBM Corp.).

## 3. Results

**Figure 1:**
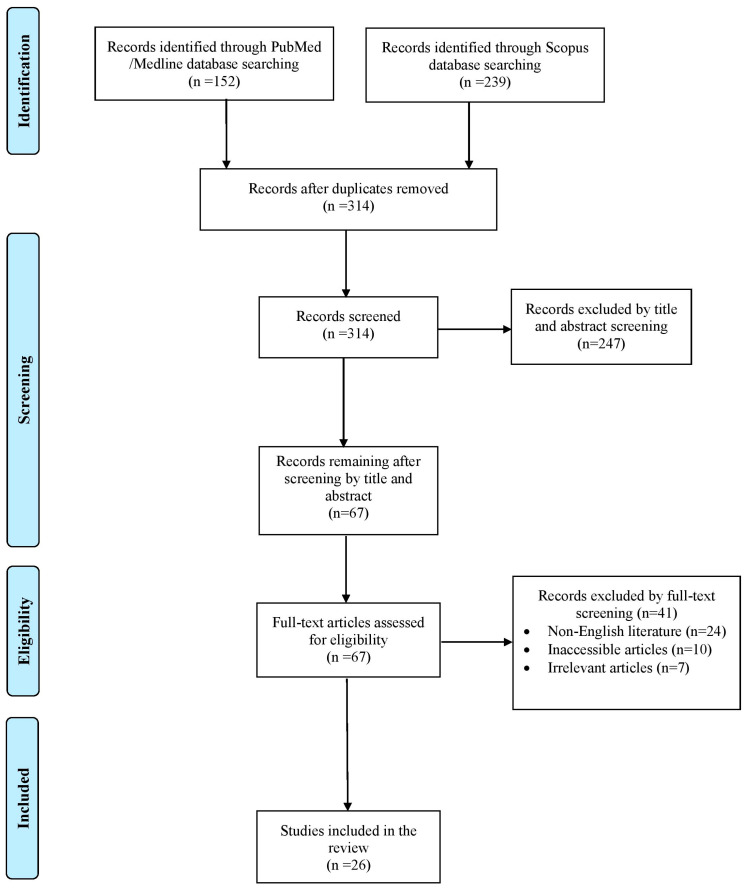
Trial flow of this review.

The literature search yielded a total of 391 studies. After the elimination of duplicate studies, record screening and the implementation of the snowball procedure, only 26 articles, published from 1933 to 2023, met the inclusion criteria and were included in this review. A graphical representation of the selection process is shown in Figure 1.

**Figure 2:**
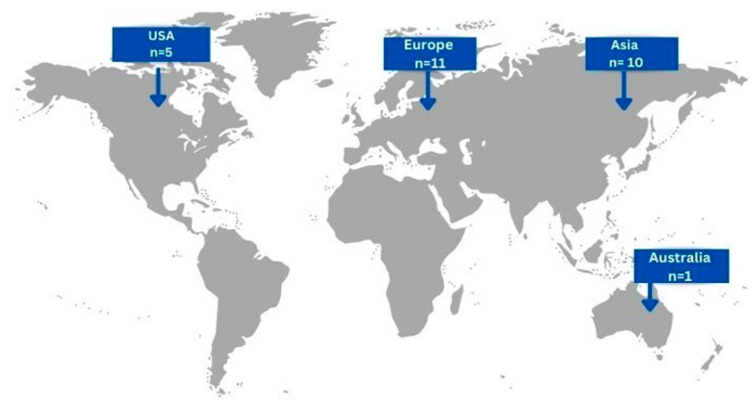
Geographical distribution of gastric actinomycosis infection worldwide (n = number of patients).

In total, the included articles concerned 27 patients, 15 males (55.56%) and 12 (44.44%) females, with a mean age of 55.11 ± 17.48 (mean, SD). Eleven cases (40.74%) originated from European countries, ten cases (37.04%) from Asian countries, five cases (18.52%) form USA and only one case (3.7%) from Australia [Figure 2]. Concerning risk factors related to the development of gastric actinomycosis, 7 patients (25.93%) had a history of abdominal surgery, 4 patients (14.81%) had a smoking history, 2 patients (7.41%) had undergone chemotherapy, and only 1 patient (3.7%) presented with a history of prolonged hospitalization. Interestingly, history of radiation and renal insufficiency were not reported as risk factors in the cases included.

The majority of patients (96.15%) complained of various symptoms, while only one patient (3.85%) was asymptomatic. Abdominal pain was present in the majority of cases (19 patients, 73.08%), while 11 patients (40.74%) complained of weight loss, 8 patients (30.77%) of nausea or vomiting, 5 patients (19.23%) of fever, 4 patients (15.38%) of gastrointestinal bleeding and the existence of a palpable mass, respectively, and 1 patient (3.85%) of diarrhea. Detailed clinical manifestations and signs of the patients included are demonstrated in Table 1. Out of the available data, the mean duration of symptoms was estimated as 7.6 ± 17.15 months (mean, SD). The maximum duration of symptoms was 60 months, while acute onset of symptoms or symptoms for less than one week was reported in 8 (36.36%) cases. In eight patients (29.62%), actinomycosis was not limited to gastric involvement. Specifically, five cases (18.51%) presented with concurrent infection of other organs; two patients developed duodenum infection, one patient peritonitis, and one patient liver infection, while another patient presented with disseminated abdominal infection involving the liver, spleen, kidneys, and the lungs. In three patients (11.1%), the infection spread through tissue continuity to the transverse colon.

Concerning the diagnostic approach for this rare entity, endoscopy was performed in 16 cases (59.26%), computed tomography (CT) in 13 cases (48.15%), ultrasonography (US) in 6 cases (22.22%), and magnetic resonance imaging (MRI) in 3 cases (11.11%). Imaging findings of the included cases are summarized in Table 2. Out of the 27 cases, 18 cases (66.66%) were diagnosed preoperatively, via endoscopy and biopsy (51.85%) or via cultures (14.81%). In total, nine cases (33.33%) were diagnosed postoperatively, with biopsies obtained after surgery (52.94%). More specifically, gastric or sputum cultures were obtained in seven cases (25.93%) and were positive in 57.14% of the cultured specimens. Amongst the patients undergoing endoscopy, 12 cases of actinomycosis were confirmed via endoscopic biopsy (75%). Initial diagnosis was misinterpreted and required further investigation in 10 cases (37.04%). All cases of gastric actinomycosis included in this study were confirmed microbiologically or histologically, with diagnosis based solely on culture or histological findings and not on imaging alone.

Regarding the therapeutic approach, out of the available data, 8 patients (32%) received antimicrobials, 6 patients (24%) underwent surgery, and 11 patients (44%) underwent both antimicrobial and surgical treatments. In total, 14 patients (77.78%) received penicillin, and the mean duration of antimicrobial treatment was 5.85 months. More specifically, penicillin alone was administered in eleven cases (61.11%), penicillin along with sulphadiazine was administered to one patient (5.56%), penicillin along with doxycycline was administered to one patient (5.56%), and penicillin combined with amoxicillin was administered to one patient (5.56%), while amoxicillin alone was administered to two patients (11.11%), ampicillin and sulbactam were administered to one patient (11.11%), and imipenem alone was administered in one patient (11.11%). Concerning the surgical approach, eight patients (47.06%) underwent subtotal gastrectomy, three patients (17.65%) underwent surgical removal of the lesion, and three patients (17.65%) underwent subtotal gastrectomy along with hemicolectomy, while two (11.76%) and one patient (5.88%) underwent exploratory laparotomy and endoscopic excision of the lesion, respectively. The most common clinical symptom among the patients subjected to surgery was abdominal pain in 12 cases (70.58%), followed by general symptoms of weight loss or anorexia in 9 patients (52.94%). Nausea or vomiting were present in six patients (35.3%) and GI bleeding in four individuals (23.52%).

In total, three patients died, two in the first month and one after 7 months. In addition, the 6-month and one-year cumulative survival rates were 13.81% (95% CI: 3.45–55.26%) and 33.81% (95% CI: 9.3–122.9%), respectively. Notably, in regard to the three lethal cases, 2 out 3 cases were male. Clinical symptoms included abdominal pain and general symptoms of fatigue or anorexia in all patients, GI bleeding and the presence of a palpable mass in one case, and diarrhea in one case. Two out of three cases had a history of abdominal surgery, but no other notable risk factors were identified. Diagnosis was established via pus cultures in one case, via histologic examination of surgical specimens in the second case, and via both endoscopic biopsy and pus culture in the third lethal case. Concerning the therapeutic approach, interestingly, none of these cases received antimicrobial treatment. Two out three patients were subjected to abdominal surgery; the first patient underwent exploratory laparotomy combined with abscess drainage and died three weeks later, while the second patient underwent subtotal gastrectomy and died 7 months later.

## 4. Discussion

Actinomycosis constitutes a rare chronic granulomatous disease caused by *Actinomyces* spp. infection. *Actinomyces* is a genus of the Actinomyceta class of bacteria; they are Gram-positive, filamentous, facultative anaerobic and nonspore-forming bacilli that are part of the normal human flora [6]. These microorganisms exist mainly in the oropharynx, urogenital tract or gastrointestinal tract and normally exhibit low pathogenicity [7]. Amongst all known *Actinomyces* species, *Actinomyces israelii*, *Actinomyces naeslundii*, *Actinomyces odontolyticus*, *Actinomyces viscosus*, *Actinomyces meyeri*, *and Actinomyces gerencseriae* are reported to cause the majority of infections in humans [6]. Infections by these opportunistic pathogens are commonly observed in immunocompromised patients or cases of disruption of the mucosal barrier allowing for direct bacterial invasion [2]. The most frequent sites of infection include the cervicofacial area (50%), followed by the abdominopelvic area (20%) and pulmonary actinomycosis [8]. *Actinomyces* infection of the gastrointestinal tract is mainly located in the cecum, appendix and colon [9]. Primary gastric actinomycosis is exceedingly rare as a result of the high luminal acidity and low gastric pH inhibiting bacterial growth [10,11]. To our knowledge, this is the first review of recorded cases regarding actinomycosis of the stomach.

Gastric actinomycosis is often related to immunosuppression induced by malignancy, HIV, chemotherapy or organ transplants. In the present review, immunosuppression was mainly noted in patients receiving chemotherapy, with a percentage of 7.41%. Older age and male sex are reported as possible risk factors that precipitate abdominal actinomycosis [6]. In this review, the majority of cases were males (55.56%). Moreover, patients with several comorbidities, prolonged medications, malnutrition or a history of abdominal surgery present a higher risk of infection [12,13]. In the present review, the interval between abdominal surgery and clinical development of the infection ranged between 6 months and 12 years. Interestingly, previous gastric operations including antrum resection are considered predisposing factors for actinomycosis by inducing gastric hypoacidity [14,15]. Foreign bodies, such as IUDs, can also potentially lead to infection by inducing mucosal traumas and facilitating bacterial invasion. Two patients were noted to have an IUD in the present review [1]. However, in the majority of cases, no risk factors were identified, and the pathogenesis of the infection remains unclear.

Clinical manifestation of gastric actinomycosis is unspecific [8]. The most predominant and initial symptom is epigastric pain, although patients might exhibit low-grade fever and weight loss as well [16]. In the present study, abdominal pain was observed in 73.08% of patients, while 19.23% developed fever and 40.74% presented weight loss. GI bleeding might also occur, either in the form of hematemesis or melena, and, in this review, it was noted in 15.38% of patients. Actinomycosis of the stomach can also present as a palpable mass in the abdominal area. The duration of these atypical symptoms is usually less than a year [14]; however, in the present review, two patients experienced symptoms of the infection for five years. Due to the particularly atypical and progressive clinical manifestation, gastric actinomycosis may mimic malignancy or several other infections such as tuberculosis or nocardiosis [10,17]. Prompt clinical suspicion is crucial for successful treatment; thus, physicians should be aware of this rare entity and include it in their differential diagnosis.

Regarding the diagnostic process for gastric actinomycosis, laboratory examinations and radiologic examinations are usually not indicative of the disease. The most common radiologic findings are peptic ulceration or malignancy [18]. Imaging features when using abdominal ultrasound are also ambiguous. Gastric wall thickening, detection of mass or enlarged lymph nodes can be observed but are not suggestive of the disease [19]. CT and MRI assist in the diagnostic process. CT findings often consist of thickening of the stomach wall, and extensive masses that may infiltrate the stomach, as well as numerous surrounding organs, such as the colon [8,20]. MRI is not as frequently applied as CT; of note, only three patients in the present review underwent MRI and, in these patients, actinomycosis appeared as a gastric lesion. The lesion can be hypointense on T1 and hyperintense on T2, have fluid density and a cystic appearance, or infiltrate adjacent tissues [6,8]. These elusive imaging features may lead to misdiagnosis; neoplasms such as adenocarcinomas or lymphomas, as well as abscesses or diffuse inflammatory conditions of the stomach, may be suspected. Interestingly, in the present analysis, ten patients (37%) were misdiagnosed; four were assumed to have tumors, while three were considered to have other inflammatory processes.

GI endoscopic techniques serve as important diagnostic modalities for gastric actinomycosis. In the current review, 59.2% of the included patients underwent upper GI endoscopy. Equivalently to other radiologic examinations, endoscopic features are usually not definitive of the infection and mainly consist of ulcerated or infiltrative gastric lesions involving the mucosa or submucosa, at times with irregular margins or yellowish exudate or necrotic material [14,19,21]. Hyperemia and edema of the mucosa may also be present [22,23]. Notably, there are cases where upper GI endoscopy may not reveal any sign of the infection, rendering the diagnosis extremely challenging [24]. In the current study, endoscopy was normal in one patient (3.7%). Upper GI endoscopy is also used as a tool to obtain biopsies from all suspicious lesions. Regarding endoscopic ultrasound (EUS), few data on imaging features of actinomycosis is available. Three (11.1%) of the included patients were subjected to EUS; the observations included a calcified hypoechoic mass arising from the submucosa in the first patient, gastric wall thickening and anechoic lesions in the second patient, and fluid collection in the third patient. An EUS image is not specific for *Actinomyces* infection since abscesses, necrosis and cancer may present similar features [14].

Histological examination of the infected tissue, and isolation of *Actinomyces* in cultures establish the final diagnosis. Histological examination is performed either preoperatively from material obtained by endoscopic biopsies or postoperatively on the resected surgical specimen. *Actinomyces* spp. infection microscopically appears as a chronic granulomatous formation, fibrosis and groups of Gram-positive filamentous bacilli with clubbed appendages composing “sulfur granules” [18,25]. In the current review, due to the heterogeneity of the microscopic findings reported, no further analysis could be obtained. Sulfur granules provide a mechanism of resistance to phagocytosis and are common histological findings in *Actinomyces* spp. infections; in general, they are observed in 75% of cases. In addition, Gram staining reveals typical Gram-positive filamentous bacteria peripherally of the sulfur granules and is more sensitive than cultures for the detection of the pathogen [1]. Infection is confirmed by microorganism isolation in cultures of pus or tissue from surgical specimen or biopsies [26,27]. However, cultures may remain sterile in more than 50% of cases as a result of previous antimicrobial administration or even inhibition of the pathogen’s growth caused by concomitant microorganisms [28]. In particular, five patients (18.5%) included in the present review had a concomitant infection and only in one of them were the cultures positive for *Actinomyces*. Moreover, cultures may require up to 20 days to indicate bacterial growth. In the present review, the cultures were positive in seven patients (25.9%) and, in four of these patients, pus was used as culture material. Given the high rate of misdiagnosis, clinicians must adopt a multimodal diagnostic approach. Upper GI endoscopy with biopsy, combined with histological examination revealing “sulfur granules” and Gram-positive filamentous bacteria, is critical for an accurate diagnosis. In cases where cultures remain sterile, molecular techniques such as polymerase chain reaction (PCR) may enhance pathogen identification, reducing unnecessary surgical interventions.

Differential diagnosis of gastric actinomycosis is demanding given that this entity presents with nonspecific clinical features, making it challenging to distinguish from other conditions such as tuberculosis, nocardiosis, and malignancies [8]. Differentiation relies primarily on histological examination and microbiological cultures. Tuberculosis often presents with granulomas on histology and is confirmed through acid-fast bacilli staining or PCR testing [10]. In contrast, nocardiosis can mimic actinomycosis but it is distinguished by modified acid-fast staining and unique culture characteristics [17]. Malignancies, particularly gastric cancers, may share similar imaging findings with actinomycosis [29]. Biopsy specimens in actinomycosis typically reveal sulfur granules and branching filamentous bacteria, which are absent in malignancies [18]. Given these diagnostic challenges, a multidisciplinary approach incorporating imaging, histopathology, and microbiology is crucial for an accurate diagnosis.

Given the challenges encountered in the diagnostic process, a surgical approach was widely chosen both as a diagnostic and treatment option through the literature. Exploratory laparotomy was performed in three patients (11.11%). Surgery in gastric actinomycosis mainly consists of total or subtotal gastrectomy, aiming to complete mass excision [24,30]. Removal of surrounding tissues involved in infectious process may also be necessary [31]. However, in general, surgical removal of the infected areas is associated with a favorable outcome [32]. Initially, intravenous and oral administration of antimicrobials is strongly recommended for a prolonged period of time (6–12 months). Penicillin remains the agent of choice to treat actinomycosis; intravenous administration is applied for 2-6 weeks, followed by oral administration for up to 12 months [16,33]. Ampicillin, macrolides, tetracyclines, clindamycin and cephalosporins are alternative antimicrobials to treat this infection; however, they are not widely administered [34,35]. In the present review, only one patient received imipenem, one patient received piperacillin/tazobactam and one received patient ampicillin and sulbactam. Given that nearly half of the reviewed cases required surgical intervention, a more conservative approach with empiric antibiotic therapy should be emphasized when histopathology strongly suggests actinomycosis. More research is required in order to clarify whether surgical resection should be reserved for complications such as perforation, obstruction, or refractory cases. Moreover, the integration of personalized medicine approaches, such as antimicrobial susceptibility testing and host immune profiling, may further optimize treatment strategies by selecting the most effective and least toxic therapeutic regimen for each patient. When diagnosed promptly and treated properly, gastric actinomycosis is related to a favorable clinical outcome; in the present study, only three patients (17.65%) died of the disease.

To the best of our knowledge, this is the first review of the literature regarding the epidemiology, clinical features, diagnostic processes and therapeutic strategy of actinomycosis of the stomach. Nonetheless, the present analysis is subjected to particular limitations. This review included only case series and case reports with adequate data whose credibility mainly relies on careful record keeping. Certain studies lacked comprehensive data, limiting the extent of our analysis to the available information. Moreover, the heterogeneity across institutions in regard to surgical techniques and record-keeping certainly affects outcomes and time-to-event analysis. Additionally, restricting our selection to English-language studies may have introduced a potential sampling bias. While this review provides valuable insights into gastric actinomycosis, several aspects require further investigation. Future research should focus on improving diagnostic methods, particularly exploring the role of molecular techniques such as PCR to enhance pathogen detection, especially in cases where cultures remain sterile. Moreover, more studies are essential to determine the most effective treatment strategies, including the potential role of conservative management with antibiotics before considering surgical intervention. Given the high variability in clinical presentation and diagnostic challenges, multicenter studies with larger sample sizes could help establish clearer guidelines for management. Exploring the impact of personalized medicine, such as antimicrobial susceptibility testing and host immune profiling, could optimize treatment regimens for better patient outcomes.

## 5. Conclusions

Actinomycosis of the stomach constitutes an exceedingly rare clinical entity with variable and non-specific clinical manifestations and imaging characteristics. Despite the rarity of this disease, clinicians should include it in their differential diagnosis, especially in immunocompromised patients or individuals who underwent abdominal surgery and present with abdominal pain, fever, or other GI symptoms. Endoscopy has proven to be useful in indicating the lesion and obtaining material for histological examination; histology and cultures verify the existence of *Actinomyces* spp. Antimicrobial administration is strongly recommended and possibly combined with surgical excision of the infected areas; penicillin remains the antimicrobial agent of choice. In recent years, advances in personalized medicine, particularly molecular diagnostics and targeted antimicrobial therapy, have emerged as promising tools for enhancing diagnostic accuracy and optimizing treatment strategies. Personalized medicine refers to an approach that tailors medical treatment to the individual characteristics of each patient, including genetic, microbial, and immunological factors. Molecular diagnostic techniques, such as polymerase chain reaction (PCR) and next-generation sequencing (NGS), can facilitate the early and precise detection of *Actinomyces* species, thereby improving diagnostic accuracy and guiding more effective, patient-specific antimicrobial therapy. By incorporating these precision-based methods, clinicians may reduce unnecessary interventions and improve treatment efficacy. However, further clinical studies and supporting evidence from the literature are required to validate the widespread application of these approaches in actinomycosis management. Prompt clinical and microbiological suspicion, early diagnosis, and suitable antimicrobial therapy remain the mainstay for managing actinomycosis.

## Figures and Tables

**Table 1:** Clinical manifestations and signs of gastric actinomycosis.

Symptom/Sign	Number of Patients	Percentage of Patients
Abdominal pain	19	73.08%
Weight loss	11	40.74%
Nausea/vomiting	8	30.77%
Fever	5	19.23%
GI bleeding	4	15.38%
Palpable mass	4	15.38%
Diarrhea	1	3.85%
Asymptomatic	1	3.85%

GI: Gastrointestinal.

**Table 2:** Imaging findings of gastric actinomycosis.

Imaging Technique	Morphology	Size Lesions
Computed tomography	• Gastric mass • irregular thickening of gastric wall • necrosis • invasion to adjacent tissues	3–16 cm
Ultrasonography	• Thickening of gastric wall • Gastric mass	3–15 cm
Magnetic resonance imaging	• Tumor like lesion • Infiltration to adjacent tissues • Cystic areas	NR
Endoscopy	• Subepithelial tumor-like lesions • Yellowish exudate • Ulceration • Edema • Necrosis • Nodular areas	1.5–5 cm

NR: Not Reported.

## Data Availability

The data presented in this study are available upon request from the corresponding author.

## Abbreviations

The following abbreviations are used in this manuscript:

IUD	Intrauterine Device
GI	Gastrointestinal
CT	Computed Tomography
MRI	Magnetic Resonance Imaging
